# Sexual violence-related pregnancies in eastern Democratic Republic of Congo: a qualitative analysis of access to pregnancy termination services

**DOI:** 10.1186/s13031-016-0097-2

**Published:** 2016-12-21

**Authors:** Gillian Burkhardt, Jennifer Scott, Monica Adhiambo Onyango, Shada Rouhani, Sadia Haider, Ashley Greiner, Katherine Albutt, Michael VanRooyen, Susan Bartels

**Affiliations:** 1 Harvard Humanitarian Initiative, Cambridge, MA USA; 2 Department of Obstetrics and Gynecology, Beth Israel Deaconess Medical Center, Boston, MA USA; 3 Brigham and Women’s Hospital, Division of Women’s Health, Boston, MA USA; 4 Harvard Medical School, Boston, MA USA; 5 Department of Global Health, Boston University School of Public Health, Boston, MA USA; 6 Department of Emergency Medicine, Brigham and Women’s Hospital, Boston, MA USA; 7 Department of Obstetrics and Gynecology, University of Illinois, Chicago, IL USA; 8 Department of Emergency Medicine, Beth Israel Deaconess Medical Center, Boston, MA USA; 9 Department of Surgery, Massachusetts General Hospital, Boston, MA USA; 10 Harvard School of Public Health, Boston, MA USA; 11 Department of Emergency Medicine, Queen’s University, Kingston, ON Canada; 12 Department of Obstetrics and Gynecology, Boston University School of Medicine, Boston, MA USA

**Keywords:** Sexual violence, Sexual violence-related pregnancy, Pregnancy termination, Democratic Republic of Congo, Unsafe abortion

## Abstract

**Background:**

Sexual violence has been prevalent throughout the armed conflict in eastern Democratic Republic of Congo (DRC). Research on sexual violence-related pregnancies (SVRPs) and pregnancy termination in eastern DRC, a context with high prevalence of sexual violence, high maternal mortality, and restrictive abortion laws, is scant but crucial to improving the overall health of women in the DRC. Understanding women’s perceptions and experiences related to an SVRP, and in particular to pregnancy termination in this context, is critical for developing effective, targeted programming.

**Methods:**

Respondent-driven sampling (RDS) was used to recruit two subgroups of women reporting SVRPs, 1) women raising a child from an SVRP (parenting group) and 2) women who had terminated an SVRP (termination group), in Bukavu, DRC in 2012. Semi-structured qualitative interviews on pregnancy history and outcomes were conducted with a systematically selected sub-group of women recruited through RDS methodology. Interview responses were translated, transcribed and uploaded to the qualitative data analysis software Dedoose. Thematic content analysis, complemented by the constant comparative technique from grounded theory, was subsequently used as the analytic approach for data analysis.

**Results:**

Fifty-five qualitative interviews (38 parenting group and 17 termination group) were completed. The majority of women in the termination group reported using traditional herbs to terminate the SVRP, which they often obtained on their own or through family, friends and traditional healers; whereas women in the parenting group reported ongoing pregnancies after attempting pregnancy termination with herbal medications. Three women in the termination group reported accessing services in a health center. Almost half of the women in the parenting group cited fear of death from termination as a reason for continuing the pregnancy. Other women in the parenting group contemplated pregnancy termination, but did not know where to access services. Potential legal ramifications and religious beliefs also influenced access to services.

**Conclusions:**

Women in this study had limited access to evidence-based safe abortion care and faced potential consequences from unsafe abortion, including increased morbidity and mortality. Increased access to reproductive health services, particularly safe, evidence-based abortion services, is paramount for women with SVRPs in eastern DRC and other conflict-affected regions.

## Background

Sexual violence has been prevalent throughout the armed conflict in eastern Democratic Republic of Congo (DRC) and has been systematically used by various armed groups to destabilize communities [1, 2]. Exposure to sexual violence – often in the form of gang rape or sexual slavery – places women at high risk of unintended pregnancies. According to a study conducted in eastern DRC in 2010, 40 % of women reported sexual violence and an estimated 17 % of those women reported resultant sexual violence-related pregnancies (SVRPs) [2]. Unintended pregnancy is associated with risks to maternal health and poor pregnancy outcomes [3, 4], and in conflict and post-conflict settings such as eastern DRC, access to comprehensive pregnancy care, including pregnancy termination and post-abortion care, is limited [5–7].

Recent studies estimate that between 8 and 13 % of maternal deaths globally are due to unsafe induced abortion [8, 9]. Regional data suggests that at least 12 % of maternal deaths in central Africa can be attributed to unsafe abortion [9]. However, stigma and restrictions associated with abortion, may result in misclassification and under reporting of abortion-related morbidity in certain contexts [8]. In the DRC, termination of pregnancy is highly restricted, and the law is generally interpreted to allow induced abortion only to save the life of the woman [10–12]. The impact of unsafe abortion and complications from an SVRP on the maternal mortality rate in DRC (estimated at 730/1000,000 live births) is unknown [13].

While there is a growing body of evidence across multiple countries showing that women who experience intimate partner violence have an increased odds of unintended pregnancies and abortion [14], research specific to pregnancy termination among women with SVRPs, and in particular data on access to termination and methods of termination, is limited. A hospital-based study in Liberia found that of women who presented with an SVRP, 59 % requested a termination upon seeking care [15]. Another study conducted in Goma, North Kivu Province, DRC, found that 87 % of women who reported an SVRP carried the pregnancy to term; however, 55 % said they were willing to terminate, 39 % said they would terminate if appropriate care was available, and 10 % attempted to terminate the pregnancy, all under unsafe conditions [16]. Research on SVRPs and pregnancy termination in eastern DRC, a context with a high prevalence of sexual violence, high maternal mortality, and restrictive abortion laws, is important to improving the overall health of women in eastern DRC. Further understanding of women’s experiences and perspectives with regards to pregnancies and pregnancy termination in the context of sexual violence is critical for developing effective, targeted programming [17]. The data presented in this paper are part of a larger mixed methods study conducted in eastern DRC to assess the experiences of women with SVRPs. This paper presents qualitative data on access to pregnancy termination and describes the methods of pregnancy termination among women with SVRPs in eastern DRC. To our knowledge, this is the first study to examine women’s perceptions of and access to abortion in this context.

## Methods

The study was conducted in Bukavu, DRC in October and November 2012. The qualitative data presented in this article were derived from a larger mixed-methods study to understand: 1) the parenting experiences of women raising children born from an SVRP, and 2) the experiences of women who terminated an SVRP. Qualitative data on perceptions and access to termination will be presented in this manuscript.

### Inclusion and exclusion criteria

For the larger mixed methods study, participants were eligible for study participation if they self-identified as a survivor of sexual violence since the start of the war (~1996), reported a pregnancy as a result of sexual violence, and were 18 years or older. Two subgroups of women with SVRPs were included in this study: 1) women currently raising children from SVRPs (parenting group), and 2) women who had terminated SVRPs (termination group). Women were included in the parenting subgroup if they reported delivery of a live born infant and were living with and caring for the child at the time of the study. Women were excluded from the parenting group if the pregnancy resulted in a stillborn infant, if the child had since died, or if the child was not currently living with or in the care of the mother. Women were included in the termination subgroup if they self-reported an induced abortion of the SVRP. Women were ineligible to participate in the study if the SVRP aborted spontaneously. The parenting and termination groups were not mutually exclusive, and women with a history of more than one SVRP were eligible to participate in both study groups provided that the inclusion criteria for each was otherwise met. All participants completed a quantitative interview, and a subset described below completed qualitative interviews.

### Participant recruitment using RDS

Participants for the larger mixed methods study were initially recruited using respondent-driven sampling (RDS), a peer-recruitment method designed to sample hard-to-reach populations [18]. RDS methodology was selected because it allows access to a hidden population that would have otherwise been difficult to access except through convenience samples [19]. This sampling approach offers advantages over convenience samples because it controls for some of the biases that are inherent in the sampling process. Under ideal conditions RDS may approximate a randomized sample [18]. Full details of the research methodology have been published elsewhere [19] and are described briefly below.

As is typical for RDS methodology, initial study participants who met study eligibility criteria were purposively selected by local partner organizations and completed a quantitative interview. After completing the interview, each initial participant was asked to recruit up to three additional eligible peers by sharing uniquely numbered recruitment vouchers. Those peers who were eligible for the study were subsequently interviewed and then asked to recruit other potential participants, and so on. A total of 852 participants (764 in the parenting group, 83 in the termination group, and 3 who met criteria for both study groups) completed quantitative interviews. Quantitative data from the parenting and the termination group have been published elsewhere [20–22].

### Qualitative study procedures

A subset of study participants were systematically selected and invited to participate in a semi-structured qualitative interview. Due to different rates of recruitment within each study group, every 20^th^ participant in the parenting group and every 5^th^ participant in the termination group were invited to participate in a qualitative interview following completion of the quantitative questionnaire. Two semi-structured interview guides, one specific to each group, were developed for this study by the research team in collaboration with local partner organizations; the interview guides were not previously used in other settings. Interview guides were initially written in English, translated into Kiswahili by a local translator and then back translated by a different translator. Translation differences were resolved by consensus with a third translator. A panel of local collaborators reviewed the translated interview guides for accuracy and was piloted prior to data collection. The interview guide corresponding to the study group (parenting versus termination) was then verbally administered in Kiswahili by female interviewers from eastern DRC, who had completed a comprehensive 6-day training on research ethics and methods. Interviews were semi-structured; participants were asked open-ended questions to obtain further information about pregnancy history, sexual violence and the SVRP, decision-making process regarding continuation or termination of the pregnancy, and concerns for the future. Interviews were conducted in private in the study office located in Bukavu. Responses were hand-recorded in Kiswahili by the interviewer, translated into English and transcribed verbatim into electronic transcripts by a local trained translator. Interviews were not audio recorded to protect participant identities.

### Coding and analysis

Thematic content analysis, which was complemented by the constant comparative technique from grounded theory, was used as the analytic approach for data analysis [23, 24]. The iterative process of concurrent data collection and analysis was not used due to study logistics and lag time in the translation of interview transcripts.

Electronic files containing the English transcripts were uploaded to the qualitative data analysis software Dedoose (Version 5.0.11, Los Angeles, CA) [25]. Documents were examined consecutively line-by-line in order to identify the thought patterns, feelings and actions that were described by participants in their interviews, while also remaining sensitive to the existing literature base as well as to being purposeful about separating relevant and non-relevant data. Two researchers initially reviewed transcripts in order to determine preliminary coding structures for organizing the data thematically. These researchers then independently coded transcripts and the data were organized into key conceptual themes. New codes were added as themes that did not fit the initial coding structure emerged. Coding inter-rater reliability was measured with a pooled Cohen’s kappa, which demonstrated a very strong agreement at 0.92 [26]. This paper presents data on the key conceptual themes of access to and perceptions of abortion; other themes will be presented in separate manuscripts.

### Positionality of researchers

Our research team is comprised of eight academic physicians and one academic nurse midwife based at U.S. academic institutions, all with significant expertise in DRC and sexual violence. Eight members of the research team worked in the DRC either during the study design, implementation, and dissemination or had prior experience working in the DRC. All members of the research team possessed a clinical and/or research background in sexual violence and/or comprehensive reproductive health in humanitarian settings. The interviews were conducted by Congolese researchers with extensive experience working with sexual violence survivors and reproductive health. Study design, implementation, and data analysis were all informed through close partnerships with three organizations in Bukavu, DRC: Centre d’Assistance Médico-Psychosociale, Acteurs Dans le Developpement et Droit a la Sante pour Tous, and Action Pour la lutte Contre L’ignorance du SIDA.

### Ethics approval and consent to participate

Human subjects research approval was obtained from Harvard School of Public Health and a community advisory board in Bukavu provided study oversight. Permission to conduct the study was also obtained from the medical inspector in South Kivu Province. Verbal informed consent was obtained due to concerns about illiteracy and concerns that written consent could identify the participant as a sexual violence survivor. No identifying information was collected. Participants who completed the study were offered a headscarf ($1 USD) as compensation for their time and transportation reimbursement (up to $8 USD) was provided directly to the taxi driver. A trained psychosocial assistant offered counseling services on-site to participants who were distressed or who requested counseling services. All participants received a referral card for medical care and/or mental health counseling.

## Results

### Demographics of participants

A total of 55 qualitative interviews were conducted, including 38 from the parenting group and 17 from the termination group. Demographics of participants are presented in Table 1. The mean age was 33.5 years ± 10.8 years (18–60 years). The mean number of pregnancies was 4.6 (range 1–12) and the mean number of living children was 3.6 (range 1–9). Approximately half (53.7 %) of women were divorced, separated or widowed, often reporting that armed groups had killed their husbands. Women may have identified as being married (20 %), even if their husbands were kidnapped, or if they were unsure whether their husbands were alive. A smaller percentage (7.3 %) of women specifically reported their marital status as husband missing. Thirty percent of women stated that they were abandoned or divorced from their spouses after they experienced sexual violence and/or became pregnant from sexual violence. Just over half (53.8 %) of the women reported that they had received no formal education, while 32.7 and 13.5 % reported any primary or any secondary school respectively. At the time the interview was conducted, 71.2 % women were living in the urban area of Bukavu, the provincial capital of South-Kivu, and the remainder reported living in rural areas; however, almost all (94.2 %) of women identified their home location as a rural town or village within the South Kivu province.

**Table 1:** Selected demographics for parenting and termination participants

Demographic characteristics of survey participants	
Characteristic	All respondents (mean (SD) or *n* (%))
Age of respondent, years	33.7
Number of pregnancies	4.6
Number of living children	3.9
Marital status (*n* = 55)	
Divorced or separated	16 (29.1 %)
Widowed	13 (23.6 %)
Married	11 (20.0 %)
Single, never married	11 (20.0 %)
Husband missing	4 (7.3 %)
Education level (*n* = 52)*	
No education	28 (53.8 %)
Any primary school	17 (32.7 %)
Any secondary school	7 (13.5 %)
Meals per day (*n* = 52)^a^	
One	38 (73.1 %)
Two	14 (26.9 %)
Religion (*n* = 52)^a^	
Catholic	28 (53.8 %)
Protestant	24 (46.2 %)
Living at the time of interview (*n* = 52)^a^	
Urban (Bukavu)	37 (71.2 %)
Rural	15 (28.8 %)
Home location (*n* = 52)^a^	
Urban (Bukavu)	47 (94.2 %)
Rural	3 (5.8 %)

^a^Demographic information incomplete for three out of the total 55 participants

### Theme 1: Methods of pregnancy termination

Of the women reporting a termination of pregnancy, most (11/17) used a traditional herb as the method for termination. Other termination methods described in the interviews included quinine (3/17), injection (1/17) and other unspecified oral medication (2/17). A traditional medicine, “*cimpokolo*”, was described repeatedly and appeared to be the most common abortifacient in this population (7/17). Women interviewed commonly reported that they obtained this medicine on their own or did not specify where it was obtained (5/17), or obtained the medicine with the assistance of a family member (2/17), relative (2/17) or a traditional healer (5/17), typically referred to as a “*wise”* or *“old”* woman.

*“I took medicine called “Cimpokolo”, I picked it and got it pounded. I pressed it in a glass and drank it. I didn’t see anybody to terminate the pregnancy because I knew since my youth that such medicine terminates pregnancies*.”

36 year-old woman with four children, widowed, who terminated an SVRP

*“When I met the wise woman, she gave me the medicine (some herbs called Cimpokolo). She mixed it with salt. I took one mug of it, double dose in the morning and in the night.”*

30 year-old woman with four children, spouse missing, who terminated an SVRP

*“My aunt pounded some herbal medicine. She gave me a glass full of it. Then I felt abdominal pain. Some black blood was running out from my vagina and then red blood after 3 days. Something like a ball fell down.”*

25 year-old woman without any children, abandoned by her spouse after the sexual assault, who terminated an SVRP

Three participants in the termination group (3/17) accessed a health care provider for assistance with the termination where they reported having received an injection (1/17) or oral quinine (2/17) to terminate the pregnancy.

*“I went to see a nurse at the Health Center. He gave me an injection. Before injection he asked me if I preferred to have oral medication or injection. I chose injection because I don’t like tablets.”*

21 year-old woman with one child, single, who was raising one child conceived from sexual violence and who terminated a second pregnancy resulting from sexual violence

*“I went to a health center to see a nurse. I told him I had abdominal troubles. After medical examination he let me know I was pregnant. I told him how I experienced sexual violence and asked his assistance to terminate it. He gave me a tablet called 050[quinine] for termination.”*

33 year-old woman without any children, spouse killed in the war, who terminated an SVRP

### Theme 2: O n-going pregnancies

Women who were raising children conceived from sexual violence (parenting group) reported that they considered terminating their pregnancies. Five (5/38) women in the parenting group specifically described on-going pregnancies after attempting to terminate.

“*I tried to terminate the pregnancy by drinking some traditional medicine made of herbs but it failed. I decided to carry the pregnancy to term after taking enough medicines in vain.”*

22 year-old woman with one child, single, raising a child conceived from sexual violence

*“I tried many times to perform termination but failed. Presently, I am pregnant from sexual violence. I am taking medicine for termination without success.”*

40 year-old woman with six children, widowed, raising one child conceived from sexual violence in 2006 and was pregnant with a second SVRP at the time of the study

A few women raising children from sexual violence reported that they had contemplated terminating the SVRP, but they did not know how to terminate (3/38). One woman also stated she refused assistance by health care providers (1/38).

*“I decided to carry that pregnancy to term because nobody told me about a good medicine.”*

45 year-old woman with five children, spouse disappeared during the war, raising a child conceived from sexual violence

*“I informed one of the [armed group] that I was pregnant from him and I wanted to terminate it….I couldn’t terminate the pregnancy when I was the bush….I didn’t know how to terminate it*.”

33 year-old woman with two children, spouse killed in the war, raising a child conceived from sexual violence

*“Yes, I intended to terminate the pregnancy but physicians refused to help me.”*

40 year-old woman with three children, widowed, raising a child conceived of sexual violence

### Theme 3: Consequences of pregnancy termination

Women raising children from SVRPs expressed concerns about potential complications related to termination of an SVRP. Almost half of the women in the parenting group (18/38) specifically cited that they were concerned they might die as a direct result of an induced abortion.

*“I informed my cousin. She was ready to help me terminate it, but I refused for fear of death. When I was about to terminate the pregnancy, another woman died of termination. I was so nervous that I decided to carry it to term.”*

23 year-old woman with one child, single, raising a child conceived from sexual violence

*“I couldn’t terminate that pregnancy because I thought it [the termination] could kill me.”*

30 year-old woman with four children, widowed, raising a child conceived from sexual violence

Women also explained that their fear of dying was heightened by that fact that they were the sole caregiver for their other children.

*“I decided to carry this pregnancy to term for fear of death…I didn’t want to abandon my children or let them become orphans.”*

48 year-old woman with six children, spouse captured by armed combatants and had not returned, raising a child conceived from sexual violence

### Theme 4: Attitudes toward pregnancy termination

Women who terminated an SVRP differentiated pregnancies conceived from sexual violence and other pregnancies.

*“Once married, to terminate a pregnancy is a big issue because the spouse is known. But to terminate a pregnancy from sexual violence is not a problem because it’s from a bandit.”*

60 year-old woman with six children, spouse missing, who terminated an SVRP

Religion influenced attitudes toward pregnancy termination. Women in both study groups, those who were raising a child conceived of sexual violence (7/38) and those who had terminated SVRPs (13/17), viewed abortion as a sin.

*“I couldn’t terminate the pregnancy because I was a Christian and I should avoid sins.”*

37 year-old woman with two children, separated from her spouse, raising a child conceived from sexual violence

*“To terminate a pregnancy is bad and it’s a sin because of killing God’s creation.”*

40 year-old woman with seven children, married, who terminated an SVRP

Five women in the study sample, one who was raising a child from sexual violence and four who had terminated a pregnancy, called on the government to change the laws allowing for legalization of pregnancy termination for SVRPs.

*“I think Congo should make termination legal and save women victims of sexual violence from any juridical case or jail sentence.”*

30 year-old with four children, spouse missing, who terminated an SVRP

*“Congolese women undergo hardships due to sexual violence…the government should authorize termination.”*

38 year-old woman with six children, abandoned by her spouse, raising a child conceived from sexual violence

## Discussion

Findings from the study highlight women’s experiences related to termination of SVRPs in eastern DRC. The qualitative data offer perspectives on the reproductive options available and accessible for women with SVRPs, as well as attitudes toward termination of SVRPs. Our findings also point to limited utilization of evidence-based methods of pregnancy termination for women with SVRPs.

The qualitative results reveal that women in this study often used herbs or medicines to induce an abortion obtained through informal health care networks, either from a friend, family member or traditional healer. The majority of women in this study who terminated the pregnancy used non-evidence based methods, most commonly a traditional herb known as *Cimpokolo*, known by the scientific name *Phytolacca dodecandra L’Hérit* [27, 28]. The qualitative finding reflect the data from the larger quantitative RDS sample, in which 31 % (26/86) of women cited *Cimpokolo* as the most common oral medication or herb used to induce abortion [22]. There is minimal evidence on the mechanism or efficacy of this traditional herb, although other herbal and plant-based abortifacients have been described in the literature [29]. Women in this study did not mention using evidence-based methods of safe abortion such as misoprostol, mifepristone, or vacuum aspiration [30]. This study did not fully evaluate if such evidence-based methods were available to women or if women were aware of these methods. Further understanding of women’s knowledge on methods of termination is warranted.

Few women in the qualitative sample accessed termination services through the formal health care sector. Of the women who terminated an SVRP, only three mentioned that the abortion was conducted at a health facility or with a health care provider. Future research should examine reasons for this. This may be reflective of the fact that in settings such as DRC, with highly restrictive abortion laws, women’s access to safe abortion services, supplies, and medications, as well as skilled providers, are lacking, and may be even further limited due to the country’s post-conflict setting [31]. A recent evaluation conducted in eastern DRC found that post-abortion care was available in one hospital and in 11 out of the 25 health centers assessed; however, none of the facilities included in this assessment openly provided safe abortion services [32]. Other research has noted that implementing agencies may omit safe abortion programming due to the politically charged nature and the associated legal complexities associated with funding restrictions for abortion programs [7, 31]. Alternatively, the findings from this study could indicate that women in this context prefer to access care in the informal health sector for abortion services or are not aware where abortion services may be available. Further research on women’s preferred point of access for termination services and the availability of evidence-based methods for pregnancy termination in both the formal and informal health care sectors is needed.

For the few women in this study who did access care through the formal healthcare sector, it was not evident from the interviews whether the health providers they saw were trained on comprehensive abortion services or whether the facilities had supplies and equipment for providing safe abortion care. In can be inferred from the medications prescribed by the health facilities (injections and quinine) that the providers were not aware of evidence-based methods to terminate pregnancy. Our narratives also revealed a pervasive awareness among the women in this setting of both fear and risk of death from termination. Although the prevalence and types of complications were not directly assessed through the qualitative methodology, the fear of complications suggests that terminations may have been carried out in unsafe conditions, and that complications were common enough so that women were aware of them. More research is needed on the magnitude of unsafe abortion and the prevalence of complications from termination of SVRPs in this context.

The qualitative data reveal the complexity of attitudes towards termination of SVRPs. The data from our study highlight the importance of religious and cultural beliefs that may influence pregnancy termination in this setting. While our study was not designed to investigate providers’ attitudes towards abortion, one woman in our study sample recounted how she was turned away from a health facility when she asked for a termination. Though not a pervasive theme in our study, providers’ attitudes towards abortion have been cited as barriers to access of care in other settings [33–36]. Other studies reveal that even with legalized abortion services, women may still seek unsafe or self-induced means of pregnancy termination as a result of stigma, financial reasons, or confusion around the law, emphasizing that efforts must be focused on improving accessibility, increasing the number of skilled providers, as well as addressing the cultural attitudes toward termination [37, 38]. A more detailed understanding of attitudes among both women and providers in this context may help to provide tailored services for women with SVRPs.

Several women in our study called on the government to legalize abortion for women with SVRPs. Currently, the legal statute for abortion services in the DRC is complex and abortion is highly restricted [10–12, 39]. The 1982 DRC Penal Code stipulates that abortions are illegal and subject to 5 to 15 years of imprisonment [40]. The DRC has ratified the *Maputu Protocol,* which stipulates that conferring states can legally provide abortion to preserve physical and mental health of the woman, in cases of fetal impairment, and in cases of rape or incest [41]. However, the women in our study who discussed legal issues called upon the government to allow abortions for SVRPs, suggesting they were unaware that pregnancy termination could be considered legally permissible in cases of rape. Evidence has shown that in countries such as the DRC, restrictive abortion laws contribute to higher rates of unsafe abortion, and therefore higher rates of maternal morbidity and mortality from abortion related complications [42, 43]. It is possible that fear of legal consequences could contribute to the selection of non-evidence based methods by the women interviewed. Since the qualitative instrument did not specifically ask about legal consequences, it is also possible this association may not have emerged in our data. Further clarification on the legal context of pregnancy termination and sexual violence in the DRC for both women and health care providers is needed, and could potentially allow for increased access to safe abortion services for women who seek termination of SVRPs [44–46].

## Limitations

The data presented here are part of a larger study, which used RDS to recruit participants. The qualitative sample reflects a systematically selected convenience sample from those recruited by RDS for the larger study. Therefore, the qualitative results in this paper represent the attitudes and experiences of those interviewed but are not generalizable. Comparison of demographics between the quantitative and the qualitative interviews indicates that married women raising children from SVRPs were under-sampled in the qualitative interviews; other differences in sampling may exist and may result in over or under representation of other groups.

Study inclusion and exclusion criteria may have affected study results. The study did not include women who gave birth to but were not raising a child from an SVRP, and their opinions are not reflected in the current data. Differences in study instruments could have contributed to information that was or was not obtained among different participants.

The questions assessed termination of pregnancies, a sensitive issue to discuss, and responses may be further influenced by a social desirability bias. Although all efforts were made in assuring fidelity to the context during translation and that local terms were translated appropriately, there may be translation and interpretation errors in this data. Additionally as the interviews were not audio-recorded due to the sensitivities of the issues discussed, some data could have been lost due to filtering by the interviewers.

Finally, as with other qualitative research, the responses may be influenced by the interviewers’ own biases and perceptions and the presence of the interviewer alone can impact the response. Efforts were made to train and hire independent interviewers who were well versed in qualitative research methods as well as the sensitivities of the subject matter and cultural norms. Additionally, there may also be interpretation bias by coders and researchers.

## Conclusions

The voices of women in this study highlight critical gaps in comprehensive safe abortion and post-abortion care for women with SVRPs. While there have been advances in reproductive health in conflict-affected settings, addressing such gaps is paramount for reducing maternal morbidity and mortality in the DRC. Future research is needed to further understand barriers to safe, evidence-based reproductive health care for women with SVRPs, and in particular for women living in conflict and post-conflict settings.

## Abbreviations

DRC: Democratic Republic of Congo
IAWG: Inter-Agency Working Group on Reproductive Health in Crises
RDS: Respondent-driven sampling
SVRP: Sexual violence-related pregnancy
